# Cross-Activity Analysis of CRISPR/Cas9 Editing in Gene Families of *Solanum lycopersicum* Detected by Long-Read Sequencing

**DOI:** 10.3390/cimb47070507

**Published:** 2025-07-02

**Authors:** Ofri Kutchinsky, Dongqi Li, Guy Assa, Asaph Aharoni, Zohar Yakhini

**Affiliations:** 1 Arazi School of Computer Science, Reichman University, Herzliya 4610101, Israel; kutchinsky.ofri@post.runi.ac.il; 2 Weizmann Institute of Science, Rehovot 7610001, Israel; dongqi.li@weizmann.ac.il (D.L.); asaph.aharoni@weizmann.ac.il (A.A.); 3 The Henry & Marilyn Taub Faculty of Computer Science, Technion—Israel Institute of Technology, Haifa 3200003, Israel; guy.assa@campus.technion.ac.il

**Keywords:** genome editing, CRISPECTOR, off-target effects, editing specificity

## Abstract

CRISPR/Cas9 genome editing holds promise for precise genetic modifications, yet off-target effects remain a concern—particularly in gene families with high sequence similarity. In this study, we present a computational framework for analyzing editing specificity and cross-reactivity in gene families using long-read sequencing data. The pipeline integrates multiplex PCR, NGS, and CRISPECTOR-based analysis to detect and quantify on- and off-target events with high sensitivity. As a use case, we applied this framework to *Solanum lycopersicum*, evaluating on-target editing in thirteen gene families and analyzing off-target cross-reactivity in five representative families. While the biological results are illustrative, the primary contribution lies in the generalizable analysis approach, which can support genome editing studies in complex plant genomes and beyond.

## 1. Introduction

In recent years, genome editing using CRISPR/Cas9 technology has garnered significant attention and interest, owing to its remarkable capabilities in enabling precise modifications in genetic material. Although the capability to achieve accurate and controlled alterations at predetermined targeted genomic locations has advanced significantly, a critical challenge remains in eliminating unwanted off-target editing resulting from cross-reactivity action. The cross-reactivity phenomenon refers to the unintended interactions that occur when molecules, such as nucleic acids, bind to or react with targets other than their intended ones. In PCR (Polymerase Chain Reaction), cross-reactivity can manifest as primer-dimer formation, where primers anneal to each other rather than to the target DNA, leading to nonspecific amplification and reduced assay efficiency [1]. Additionally, cross-hybridization occurs in microarray-based assays [2,3] and CRISPR experiments, where nucleic acid probes or guides RNAsbind to off-target sequences, resulting in false measurements or off-target editing [4,5]. These occurrences highlight the critical need for advanced computational tools and continued research to accurately predict, measure, and mitigate off-target effects, thereby enhancing the reliability of gene editing and molecular assays.

Gene families are known to be prone to such off-target activity. The term gene family refers to a collection of genes sharing common functionalities, arising from gene duplication events originating from a solitary ancestral gene. These duplicated genes maintain similar sequence and structure and therefore constitute potential off-target sites [6].

The task of avoiding and measuring off-target activity may become even more difficult in plants due to properties of their genomic structure. Plants such as rice, *Populus trichocarpa*, and many others have been found to have a greater number of genes than humans, as we know today. Furthermore, plants are more evolutionarily diverse and include a wide range of species with different adaptations to various ecological niches. To thrive in different environments, plants have evolved a variety of specialized genes that allow them to respond to different factors, produce unique secondary metabolites, and adapt to diverse conditions. Plants also undergo small-, large-scale, and whole-genome duplications more frequently than animals. All of these factors contribute to the fact that plants usually have more gene families, and in most of the families, there are more members of genes in plants than in humans [7].

Given these complexities in plant genomes—such as gene duplication and high sequence redundancy—we selected *Solanum lycopersicum* as a model species. We analyzed thirteen gene families chosen for their biological relevance and sequence similarities. These families include genes encoding ethylene response factors (ERFs), UDP-glycosyltransferases, LOB domain-containing proteins, C2H2-type zinc finger proteins, auxin response factors (ARFs), and others. A complete description of all analyzed families, including guide sequences and targeted genes, is provided in Appendix A, Table A1.

Long-read sequencing offers critical advantages when studying gene families. Due to their high sequence similarity, family members often produce ambiguous alignments when analyzed with short reads, complicating the assignment of editing events to specific loci. Long reads enable full-length amplicon coverage, preserving the genomic context and allowing accurate discrimination between on- and off-target edits across homologous genes [8,9]. This is particularly important in plants, where genome duplication and structural complexity are common [10].

Various tools supporting editing activity measurement have been developed in order to better understand gene editing specificity and cross activity, as well as factors affecting off-target activity. These tools include CRISPREsso 2 [11], ICE [12], DeepCRISPR [13], etc. However, these tools primarily focus on short-read sequencing and on-target indel analysis. A key component of our approach is the use of CRISPECTOR (v2.0 ) [14,15], a tool designed to detect both on- and off-target events with high sensitivity. Unlike CRISPResso2 and ICE, which are limited in their ability to detect low-frequency off-targets, CRISPECTOR incorporates statistical modeling to identify unintended modifications. This makes it particularly well-suited for studying gene families, where sequence homology increases the likelihood of cross-reactivity. DeepCRISPR is a tool that employs deep learning for off-target prediction but does not provide experimental validation. By combining long-read sequencing with CRISPECTOR, our method enables a more comprehensive analysis of editing specificity in plant genomes.

As discussed above, the combination of high gene family similarity, frequent genome duplications in plants, and the limitations of short-read data creates significant challenges for accurately detecting off-target CRISPR/Cas9 activity. While several tools support general off-target analysis, few provide dedicated solutions for evaluating cross-reactivity within gene families—a challenge especially relevant in plant genomes.

In this work, we introduce a framework for analyzing long-read sequencing data to infer editing activity in pre-determined sites. Further, we provide an approach to using the above framework as part of an analysis of on- and off-target activity in gene families, including a scheme for setting up the data for analysis by CRISPECTOR. Finally, we demonstrate the use of our methods to measure cross-activity in gene families from Tomato seedlings (*Solanum lycopersicum* cv. M82), highlighting its potential for improving genome editing specificity in plants.

## 2. Materials and Methods

### 2.1. Plant Material and Generation of Genome-Edited Hairy Roots

Tomato seedlings (*Solanum lycopersicum* cv. M82) were grown in controlled conditions with a temperature of 24 °C and a photoperiod of 16/8 h light/dark. The genome-edited hairy roots were generated following a protocol as reported in Ron et al. [16].

Briefly, tomato seeds were sterilized in 70% ethanol for 3 min, followed by 3% commercial bleach (original concentration: 6%) for 15 min. The seeds were then washed six times with sterile distilled water and germinated on 1/2 MS medium (2.25 g/L M0222, Duchefa, Haarlem, The Netherlands, 1.5% sucrose, and 0.8% plant agar, Duchefa, Haarlem, The Netherlands, pH 5.8) in Magenta boxes. After 7–10 days under controlled tissue culture conditions, the cotyledons were excised and placed onto co-cultivation medium (4.5 g/L M0222, 3% sucrose, 0.8% plant agar, and 200 µM acetosyringone, pH 5.8) for 1 day in the dark.

The cotyledons were then immersed in an *Agrobacterium rhizogenes* (strain ATCC 15834) suspension prepared in liquid MS (4.5 g/L M0222, 3% sucrose, and 200 µM acetosyringone, pH 5.8) for 15 min with gentle shaking. The bacterial culture was used at OD600 = 0.5. A mock treatment was performed using *A. rhizogenes* competent cells without plasmid constructs to serve as a control.

**Figure 1:**
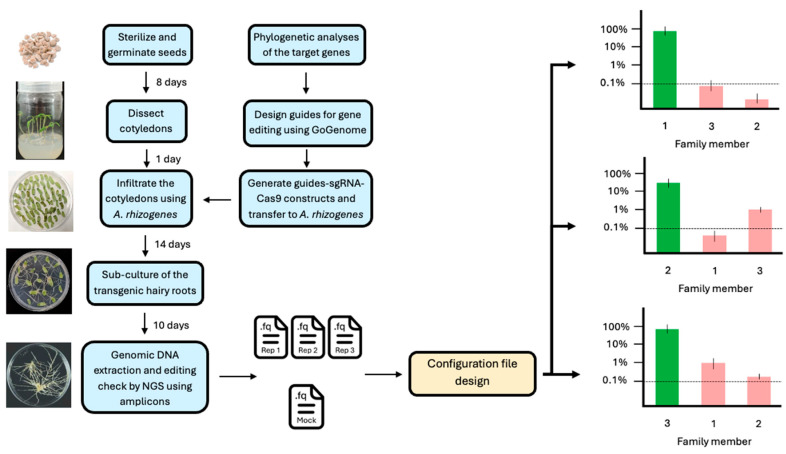
Analysis of gene families in *Agrobacterium rhizogenes* as an example of the pipeline utilizing CRISPECTOR for long-read sequencing data. The core of the pipeline is the generation of an adequate configuration file.

The cotyledons were then transferred onto co-cultivation medium plates with a layer of sterilized filter paper and incubated in the dark for 2 days. Subsequently, the cotyledons were transferred to selection medium (4.5 g/L M0222, 3% sucrose, 0.8% plant agar, pH 5.8, supplemented with 250 mg/L cefotaxime and 50 mg/L kanamycin) and maintained under controlled tissue culture conditions. The presence of the kanamycin resistance gene in the construct allowed for selection, as only successfully transformed roots were able to grow on kanamycin-containing medium. After 10 days, hairy roots emerged from the cut sites of the cotyledons. An overview of the experimental and analytical pipeline used in this study is shown in Figure 1.

### 2.2. Generation of the Constructs for Genome Editing

We generated constructs for genome editing as described in Ma et al. [17]. Briefly, one specific guide targeting the coding region of each gene family member was selected using GoGenome, https://gogenome-ui.CRISPRil.com/ (accessed on 16 March 2022). Through a two-step PCR-based method, the sgRNAs were individually inserted into an expression cassette driven by the AtU3d, AtU3b, AtU6-1, or AtU6-29 promoter in an intermediate plasmid. The resulting expression cassettes were then assembled into the w1-35Spro-GFP-Cas9-CCD binary plasmid using Golden Gate ligation cloning [18].

The plasmid was introduced into *Agrobacterium rhizogenes* via electroporation. For this, 50 µL of *A. rhizogenes* electrocompetent cells were thawed on ice, and 1 µL of plasmid DNA (100 ng/µL) was added and mixed thoroughly. The mixture was transferred to a chilled 1 mm cuvette, ensuring that the cells settled at the bottom, and electroporated using a Bio-Rad Gene Pulser (Bio-Rad, Hercules, CA, USA) at 2.0 kV. Immediately after electroporation, 1000 µL of LB medium was added, mixed well, and transferred to a 1.5 mL Eppendorf tube for recovery at 28 °C for 4 h. The culture was then spread on a selective LB plate and incubated at 28 °C for 4 days to allow colony formation.

### 2.3. Multiplex PCR NGS Assay

To assess on-target editing efficiency, we prepared SMRTbell libraries using the PacBio Barcoded Overhang Adapters 8A kit (101-628-400, PacBio, Menlo Park, CA, USA) for multiplexed amplicon sequencing of long reads. Forward primers for the amplicons were designed approximately 500 bp upstream of the target sites, while reverse primers were placed ~500 bp downstream.

Genomic DNA was extracted from the hairy roots using the cetyltrimethylammonium bromide (CTAB) method, following the protocol described by Murray and Thompson [19]. The extracted genomic DNA was used as a template for PCR amplification, performed using primers specific to the target sites and Q5^®^ High-Fidelity DNA Polymerase (M0492S, NEB, Ipswich, MA, USA). The amplified products were purified using the AMPure PB Bead Kit (100-265-900, PacBio, Menlo Park, CA, USA) to remove PCR reagents, buffers, primer dimers, and short nonspecific PCR products. Following purification, the concentration of all amplicons was measured, and equimolar amounts were pooled.

Each amplicon was prepared with six replicates treated with *Agrobacterium rhizogenes* harboring the CRISPR construct, along with one replicate treated with *A. rhizogenes* competent cells as a control, yielding a total of seven pools. Barcoding was performed using the Barcoded Overhang Adapter Kit–8B (PN 101-628-500, PacBio, Menlo Park, CA, USA), following the manufacturer’s protocol. The seven adapter ligation reactions (~16.3 μL each) were pooled into a single 1.5 mL Eppendorf tube, mixed thoroughly, and purified using AMPure PB beads.

The purified pooled library was prepared for sequencing using the SMRTbell Express Template Prep Kit 2.0 (100-938-900, PacBio, Menlo Park, CA, USA). Sequencing primers, Sequel^®^ Polymerase 3.0, dNTPs, DTT, and binding buffer were added to the barcoded amplicons, mixed well, and incubated at 30 °C for 1 h to form Polymerase-Bound SMRTbell^®^ Complexes. The complexes were cleaned using AMPure PB beads before sequencing.

For sequencing preparation, a two-step 1:100 dilution of the internal control stock was performed using Sequel^®^ Complex Dilution Buffer. A mixture of 67.9 µL of Sequel^®^ Complex Dilution Buffer, 4.8 µL of the prepared sample, 2.8 µL of twice-diluted internal control complex, 8.5 µL of DTT, and 1 µL of Sequel Additive was prepared, mixed thoroughly by finger tap, spun down for 1 s, and loaded onto the PacBio sequencing platform. For detailed information regarding raw sequencing data, amplicon sequences, primers, and gRNA guides, see Supplementary Materials.

### 2.4. Setting up CRISPECTOR for Gene Family Analysis by Preparing Configuration Files

Let F be a gene family containing n amplicons denoted as $F_{1},\ldots ,{\;F}_{n}$. Let $g_{1},\ldots ,\;g_{n}$ be the gRNA sequences designed for the editing of $F_{1},\ldots ,\;F_{n}$, respectively.

For each amplicon $F_{i},$ *i* = *1*, …, *n*: 

For each amplicon $F_{j}\;(\;j\;\neq \;i)$:

1. Collect all 20 base pairs’ long subsequences adjacent to any PAM sequence, in both forward and reverse sequences.
2. Select the subsequences with alignment score over some user-defined threshold with respect to $g_{i}$. These are determined by the Bio.pairwise2 module (Biopython v1.85).
3. Further refine the set of subsequences and keep those with an edit distance under some user-defined threshold with respect to $g_{i}$.

Let L (i, j) be the resulting list. This is the list of all potential off-target subsequences in $F_{j}\;(\;j\;\neq \;i)$ with respect to $g_{i}$. Denote the length of the list L (i, j) by k (i, j) and $k\left(i\right)=\max_{j}k\;(i,\;j)\;$.

#### Example

**Figure 2:**
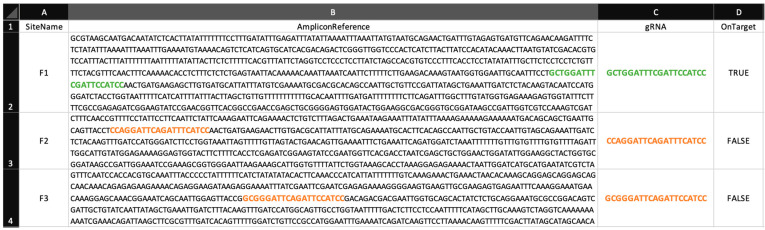
CRISPECTOR configuration file for gene family analysis. On-target sites are highlighted in green, while the putative recognition sequences are highlighted in orange.

For the gene family consisting of three genes and three amplicons, we are interested in analyzing the off-target activity of CRISPR targeted to gene 1, in genes 2 and 3. The putative recognition sequences from both amplicons 2 and 3 will be entered into the “gRNA” cells, respectively, as shown in the Figure 2.

## 3. Results

### 3.1. On-Target Activity

**Figure 3:**
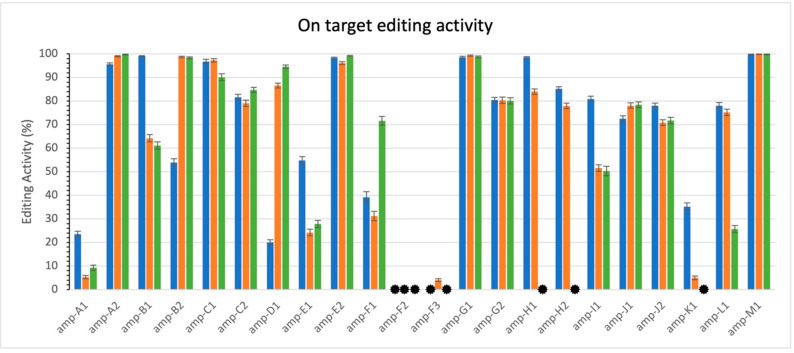
On-target editing activity. The 3 colors represent the 3 replicates. Each replicate spans all sites. Error bars are calculated by CRISPECTOR. Black asterisk (. 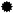) indicates values lower than 0.3%. Note variability of activity levels.

In this study, we initially investigated thirteen gene families within the *Solanum lycopersicum* genome, profiling editing activity for one to three family members in each. After quality filtering, we selected five families for deeper investigation, as listed below. Figure 3 illustrates the measured activity levels at the on-target sites (see methods 0). Notably, most sites exhibited high activity levels in at least one replicate. However, variability was observed across replicates: while some sites showed consistent activity, others displayed no activity in one replicate but high activity in others. For example, this variability is evident in amplicons H1 and H2. These results serve primarily as confirmation that CRISPECTOR successfully processes long-read sequencing data and that measurable editing activity is present. They also support our decision to focus subsequent cross-family analysis on five families with consistently observed on-target editing. Table 1 provides an overview of the on-target locations measured for the five selected families. For reference, a complete summary of all 13 gene families, including their gene members and characteristics, is provided in Appendix A, Table A1.

### 3.2. Off-Target Activity in Families

**Figure 4:**
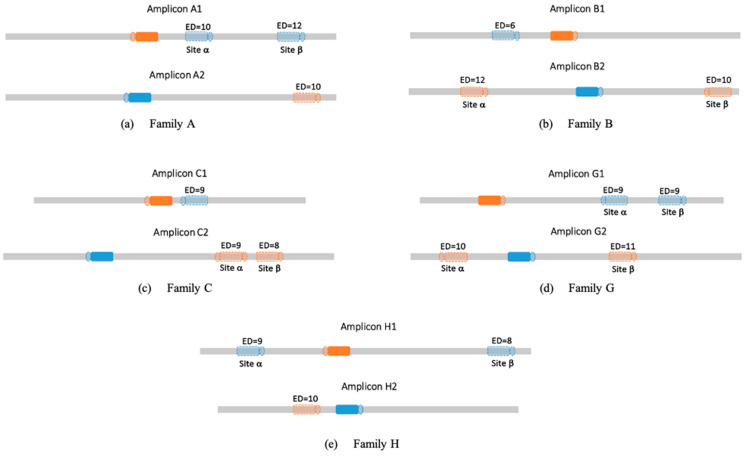
On-target and potential off-target sites. The amplicons measured in this study all contain intended (on-target sites) as well as off-target sites, since these are gene families. Full shapes represent on-target sites, hollow shapes represent the selected potential off-target sites (denoted α and β when applicable), and ED indicates the edit distance between the genomic sequence and the relevant guide (see methods 0). PAM sequences are depicted as circles adjacent to the potential off-target sites. Colors are used to match each off-target site to its corresponding on-target sequence (e.g., blue hollow shapes correspond to the blue solid on-target site in the other family member).

**Figure 5:**
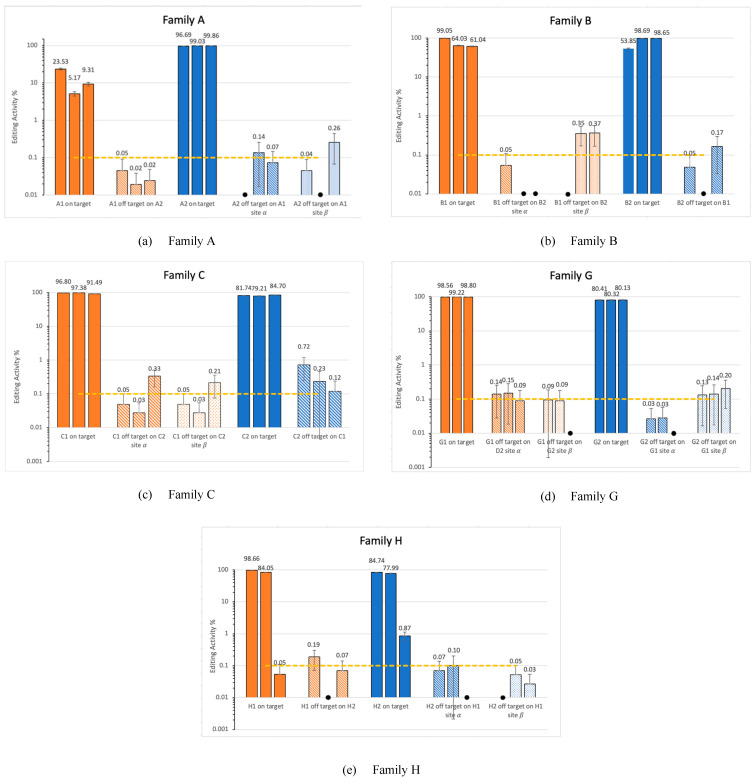
Off-target activity report. Solid colored bars represent on-target sites, while patterned colored bars represent the selected potential off-target sites. Sites α and β are as described in Figure 2. Results come with 0.95 CIs as produced by CRISPECTOR calculations. Black asterisk (. 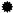) indicates values lower than 0.01%. This figure represents the results from all three replicates. The dashed line marks the 0.1% threshold, a standard benchmark in CRISPR off-target analysis.

The process described (see methods 0) allows us to analyze cross-reaction within the investigated families. For each targeted genomic location, we computed potential off-target sites from parallel family members as presented in Figure 4. Figure 5 shows the results from running CRISPECTOR on the setup described below. In total we observe some significant off-target activity in some of the families. Note, in particular, that no off-target activity at a level greater than 1% was observed in our experiment. However, we do observe significant 0.1% activity in sites with large edit distances. For example, in family B, at site β amplicon B2 we observe an activity of around 0.36% while the edit distance is 10. Similarly, in family C, amplicon C1, we observe an average activity of 0.35% with an edit distance of 9. These findings highlight the utility of the proposed framework in detecting off-target activity across homologous loci, even when sequence editing distance is substantial. While the observed activity levels are low, their consistent detection at distant sites suggests the potential for weak cross-reactivity within gene families that may go undetected with traditional short-read or alignment-filtered approaches. This illustrates the strength of combining long-read sequencing—enabling accurate assignment of editing events to specific loci—with statistical off-target detection in gene family contexts, especially when aiming to support safer or more precise editing designs.

## 4. Discussion

The primary focus of this study is the development of a computational framework for assessing cross-reactivity in CRISPR/Cas9 genome editing between members of gene families. This challenge arises due to sequence homology among paralogs, which can lead to unintended editing at off-target sites within the same gene family. Our framework integrates multiplex PCR, long-read amplicon sequencing, and CRISPECTOR-based analysis to enable detection of both on- and off-target activity across closely related genomic loci. From a practical perspective, the pipeline also enables the use of tools originally developed for short-read sequencing to analyze long-read sequencing data.

Our use case in *Solanum lycopersicum* demonstrates the potential of this approach. We initially examined thirteen gene families, including ERFs, UDP-glycosyltransferases, LOB domain-containing proteins, C2H2-type zinc finger proteins, and ARFs. Based on observed on-target activity, five of these families were selected for further analysis. These families exhibited diverse patterns of off-target activity, including low-frequency editing at sites with substantial edit distances from the designed guide. While the frequencies were low, their consistent detection suggests weak but reproducible cross-reactivity between closely related family members. This reinforces the need for locus-level validation in gene-dense or duplicated genomic regions.

The cross-reactivity patterns observed in this study underscore the importance of guiding RNA design when targeting gene families with high sequence similarity. By identifying unintended activity across homologous loci, the proposed framework can support future efforts to refine gRNA selection strategies and reduce off-target effects through data-driven design improvements.

Previous studies have reported off-target effects in CRISPR/Cas9 experiments, par-ticularly in plants with large and complex genomes. For example, research in rice and Arabidopsis has demonstrated that CRISPR/Cas9 can induce off-target modifications due to partial sequence complementarity with unintended genomic regions [7,20]. Similarly, Assa et al. [15] reported allele-specific editing activity in different chromosome copies of Musa acuminata, highlighting the potential for different CRISPR/Cas9 targeting within polyploid genomes. In tomato, CRISPR/Cas9 editing has been shown to produce unin-tended mutations; for instance, Jacobs et al. [21] identified unintended mutations in tomato gene families caused by sequence homology and reference genome inaccuracies, reinforcing the importance of experimental validation of editing specificity.

While this study focuses on tomato, the framework itself is generalizable. Its components—targeted amplification, long-read sequencing, and statistical analysis—are adaptable to any genome with a reference assembly and the ability to design guides and primers. In particular, plant species with large, polyploid, or repetitive genomes may benefit from this framework, which provides more accurate assignment of editing events in regions where conventional short-read methods may fail.

Our study also contributes to a deeper understanding of gene editing precision in plant systems. By utilizing long-read sequencing technologies, one can analyze editing activity across gene families, potentially providing valuable insights into the factors influencing off-target effects. Our framework offers a foundation for future studies addressing these challenges.

However, it is important to acknowledge the limitations of our study, such as the focus on a single plant species and a limited number of gene families. Future research can aim to expand the scope to include a wider range of plant genomes and gene families, demonstrating applicability in these broader contexts.

## Figures and Tables

**Table 1:** Gene families’ information. On-target sequences for the five investigated families.

Family	Members’ Guide Sequence	Amplicon Length bp
A	A1: TCTTCATCTCCAGTAAGCCT	1012
	A2: TCCGGTACGCGAAACAAGGG	1009
B	B1: GCGGCTTCCACGGCACCCAA	956
	B2: GTAACAACATTTCCAGCACA	975
C	C1: TGCTAGGAAGACAGTAGCGT	907
	C2: TGAGATTGAGAAGCCCCGCA	1100
G	G1: GCAAAGGCCAGCAGCAGCTT	1021
	G2: ATCGCGTAGCATCGTCATGG	1112
H	H1: TTTGGTTGCAGATTGAACAA	1061
	H2: CGAGTTTACTACTTTCCACA	964

## Data Availability

The original contributions presented in this study are included in the article/Supplementary Materials. Further inquiries can be directed to the corresponding author.

## Supplementary Materials

The following supporting information can be downloaded at: https://www.mdpi.com/article/10.3390/cimb47070507/s1.

## Appendix A

This appendix presents an extended table containing information on all 13 gene families initially analyzed. It includes both the five families selected for further analysis, as shown in the main text, and the additional eight families examined in the initial phase of the study.

**Table A1:** Extended summary of all 13 gene families initially analyzed, including the 5 families selected for further analysis and the additional 8 families examined in the first phase of the study.

Family	Members’ Guide Sequence	Amplicon Length bp	Targeted Gene	Gene ID
A	A1: TCTTCATCTCCAGTAAGCCT	1012	Ethylene Response Factor D.3	*Solyc01g108240*
	A2: TCCGGTACGCGAAACAAGGG	1009	Ethylene Response Factor D.4	*Solyc10g050970*
B	B1: GCGGCTTCCACGGCACCCAA	956	UDP-glycosyltransferase 75C1	*Solyc09g092500*
	B2: GTAACAACATTTCCAGCACA	975	UDP-glycosyltransferase	*Solyc12g098590*
C	C1: TGCTAGGAAGACAGTAGCGT	907	LOB domain-containing protein 40	*Solyc05g009320*
	C2: TGAGATTGAGAAGCCCCGCA	1100	LOB domain-containing protein 41	*Solyc03g119530*
D	D1: TTCACTGTGCTGCAGCTGGT	982	Zinc finger transcription factor 39	*Solyc05g052570*
E	E1: GTCATCGTCGTTTTCTGAAG	972	C-repeat binding factor 1	*Solyc03g026280*
	E2: CAATCACTACTCCCCTAATG	1116	Dehydration-responsive element-binding transcription factor	*Solyc03g124110*
F	F1: GGATGGAATCGAAATCCAGC	987	NAC domain-containing protein	*Solyc11g017470*
	F2: ACTGGTGCGGATAAGCCGAT	1207	NAC domain-containing protein	*Solyc06g060230*
	F3: TATCTCTGCAGGAAATGCGC	1101	NAC domain-containing protein	*Solyc04g009440*
G	G1: GCAAAGGCCAGCAGCAGCTT	1021	C2H2-type zinc finger protein	*Solyc04g077980*
	G2: ATCGCGTAGCATCGTCATGG	1112	C2H2-type zinc finger protein	*Solyc12g088390*
H	H1: TTTGGTTGCAGATTGAACAA	1061	Auxin Response Factor 9B	*Solyc08g008380*
	H2: CGAGTTTACTACTTTCCACA	964	Auxin Response Factor 18	*Solyc01g096070*
I	I1: GAAGCTGTCACCCACGGTGG	1059	Zinc finger protein	*Solyc01g107170*
J	J1: GCTGGGTACTTTTGATACGG	1126	Ethylene Response Factor F.4	*Solyc07g053740*
	J2: TACTTTCGATACTGCGGAGG	996	Ethylene Response Factor F.5	*Solyc10g009110*
K	K1: TCCCCAAATCAAGAATCTGC	1002	syntaxin-121-like	*Solyc10g081850*
L	L1: GTTTCCGAATCTCAACGACC	924	LOB domain-containing protein 38	*Solyc01g107190*
M	M1: GGAACATCCCCAGCAAATGG	948	Multidrug resistance protein ABC transporter family protein	*Solyc01g079550*
